# Special Issue “Bioactive Compounds in the Prevention of Chronic Diseases”

**DOI:** 10.3390/ijms27188116

**Published:** 2026-09-12

**Authors:** Diego A. Moreno, Antonio J. Ruiz-Alcaraz, Paola Maycotte

**Affiliations:** 1 Laboratorio de Fitoquímica y Alimentos Saludables (LabFAS), CSIC, CEBAS, Campus Universitario de Espinardo, 30100 Murcia, Spain; dmoreno@cebas.csic.es; 2 Department of Biochemistry, Molecular Biology and Immunology, School of Medicine, University of Murcia, Regional Campus of International Excellence “Campus Mare Nostrum”, 30120 Murcia, Spain; 3 Innate Immunity in Health and Disease, Biomedical Research Institute of Murcia (IMIB) Pascual Parrilla, 30120 Murcia, Spain; 4 Centro de Investigación Biomédica de Oriente, Instituto Mexicano del Seguro Social, 74360 Puebla, Mexico

## 1. Introduction

Bioactive compounds are naturally derived molecules with biological effects that have been used for the treatment of several diseases, including those that are highly prevalent, like cardiovascular disease, cancer, metabolic and inflammatory disorders. These compounds and their known metabolites (including, but not limited to polyphenols, fatty acids, proteins, dietary fibers, etc.) play a crucial role in the modulation of inflammation and oxidative stress, which are key factors in chronic disease development. Accordingly, most bioactive compounds are known for their antioxidant effects but can also directly modulate several signaling pathways involved in inflammation and disease, such as the NF-kB, MAPK, or COX pathways [1], or with potent antimicrobial or enzyme-modulating activities [2].

The chemical composition of natural products is diverse, with those compounds like polyphenols, flavonoids and stilbenes known to be bioactive due to their antioxidant effects but also with other compounds such as lipids or vitamins with additional described effects on the prevention and treatment of human disorders [3]. The search for safer and more effective therapeutics for life-threatening diseases is ongoing, and since many of the drugs currently in the market are of a natural origin [4], it is important to continue evaluating new bioactive molecules for drug discovery. In this Special Issue, manuscripts explore botanical extracts, formulations and natural compounds and evaluate their effects on highly prevalent diseases like cancer, kidney disease, myocarditis, cardiovascular disease, antibiotic resistance and inflammation.

### 1.1. UHPLC-QTOF-MS Profiling of Chemical Constituents in POW9TM Cocktail with Antioxidant and Anti-Proliferative Potentials Against Vero, MCF-7 and MDA-MB-231 Cells

The manuscript by Taworntawat et al. [Contribution 1] presented a detailed analysis of the patented botanical preparation POW9™, which comprised extracts of 9 medicinal plants (*Curcuma longa* L., *Cinnamomum verum* J. Presl., *Phyllanthus emblica* L., *Ginkgo biloba* L., *Centella asiatica* L. Urb., *Piper nigrum* L., *Camellia sinensis* L. Kuntze, *Garcinia mangostana* L., *Hibiscus sabdariffa* L.) complemented with basic colorimetric analyses (e.g., TPC, TFC, TEAC) for free radical scavenging capacities and obtained some potency of interest (cocktail DPPH IC50 value of 1.66 mg/mL). This was combined with innocuousness on cell study and a low intrinsic toxicity in non-malignant cells under their experimental conditions. Nevertheless, the antiproliferative effect in breast cancer cell models showed some interesting data, again in a range of concentration that can be considered excessive for translating the results in further preclinical work (e.g., MCF-7 cells, IC50 (72 h): 7.81 mg/mL; triple-negative MDA-MB-231 IC50 (72 h): 18.08 mg/mL ca.). The rather low potential bioactivity may be the consequence of additive or synergy mechanisms between the several components of the cocktail, including some lipid derivative compounds or essential oils. Breast cancer, especially the triple-negative subtype (TNBC), represents a clinical challenge because of the therapeutic resistance and systemic toxicity of the current available treatments, and therefore, natural alternatives such as the POW9™ as coadjutants of the available therapies could be part of complementary strategies, even though reducing dosage for a more physiologically relevant effect remains unsolved. This study established (in vitro) some selectivity in antiproliferative capacity with low toxicity at high dosages, the basis for further developments. The report identified several additional limitations for the future developments, not only because of the in vitro results or the need for further mechanistic investigations.

### 1.2. Anti-Inflammatory Effects of Alpha-Lipoic Acid Modulate Cystathionine-γ-Lyase Expression in RAW 264.7 Macrophages

In the manuscript by Shahid et al. [Contribution 2], the authors evaluated the time-dependent anti-inflammatory and antioxidant potential of alpha-lipoic acid (ALA), an organosulfur compound, and its mechanistic link with cystathionine-γ-lyase (CSE) expression in lipopolysaccharide (LPS)-stimulated RAW 264.7 macrophages. The authors analyzed both prophylactic (1 h pre-treatment) and therapeutic (1, 3, and 6 h post-treatment) interventions, selecting 1000 µM as the concentration for further experiments. LPS stimulation significantly increased the levels of pro-inflammatory cytokines and chemokines (TNF-α, IL-6, MCP-1), oxidative stress markers (MDA), and CSE protein expression, while markedly decreasing catalase (CAT) activity and disrupting macrophage cellular morphology. ALA treatment effectively attenuated these pro-inflammatory mediators, restored CAT antioxidant activity, reduced lipid peroxidation, and preserved macrophage morphology in a time-dependent manner, with the highest efficacy observed during pre-treatment and early post-treatment (1 h). Western blot analysis revealed that the protective effects of ALA were accompanied by the significant downregulation of CSE expression, the enzyme responsible for H_2_S gas transmitter synthesis in macrophages. Although this study provides important novel insights into the temporal dynamics of ALA in mitigating inflammation, it presents some limitations, such as the lack of pharmacological CSE inhibitors (e.g., PAG) or gene silencing/deletion models (siRNA or CSE-/-), which can be used to confirm the existence of a direct causal relationship, as well as the use of high in vitro doses. Therefore, future pharmacokinetic, in vivo disease models and target validation studies are granted to further elucidate this pathway and support translational applications for the clinical management of inflammatory conditions like sepsis.

### 1.3. Brassica Extracts Prevent Benzo(a)pyrene-Induced Transformation by Modulating Reactive Oxygen Species and Autophagy

In the manuscript by Montes-Alvarado et al. [Contribution 3], the authors evaluated the chemopreventive potential of aqueous broccoli sprout extract (BSE) and red cabbage leaf aqueous extract (RCA) against carcinogen-induced cellular transformation in the non-tumorigenic human mammary epithelial cell line MCF10A. Both extracts were characterized by high levels of glucosinolate hydrolysis products, including isothiocyanates (sulforaphane and iberin) and indole-3-carbinol, with RCA displaying an overall higher concentration of these bioactive analytes. Using benzo(a)pyrene (B(a)P), an environmental polycyclic aromatic hydrocarbon and known group I carcinogen, the authors established an in vitro transformation model characterized by transient intracellular reactive oxygen species (ROS) accumulation, early metabolic shifts (observed by enhanced MTT conversion), and increased long-term clonogenic proliferation. Treatment with BSE (150 µg/mL and 1 mg/mL) or RCA (10 µg/mL and 25 µg/mL) successfully prevented the B(a)P-induced metabolic alterations, long-term hyperproliferation, and ROS elevation without altering baseline cell viability or morphology. Furthermore, autophagic flux assessment via LC3-II turnover in the presence of chloroquine demonstrated that RCA, but not BSE, significantly induced autophagy, a relevant homeostatic and tumor-suppressive pathway, possibly due to the presence of additional bioactive compounds in RCA such as anthocyanins. While this study provides compelling preclinical evidence supporting the potential chemopreventive effects of *Brassica*-derived compounds against carcinogen-induced cellular transformation, further mechanistic studies are needed to determine the precise downstream molecular targets (such as NRF2 activation or CYP450 inhibition) and to validate these chemopreventive effects in complex in vivo models.

### 1.4. Phytochemicals from Purwoceng (Pimpinella pruatjan) and Their Potential in Chronic Disease Prevention: Focus on Kidney Health

In the manuscript by Dewi et al. [Contribution 4], the authors evaluated the potential of purwoceng (*Pimpinella pruatjan*), a native plant from Indonesia known for its vasodilatory effects, in the prevention of chronic kidney damage. The purwoceng root extract was rich in phenolic, flavonoid compounds and fatty acids, with juniperic acid being the most abundant compound and with limited intrinsic antioxidant activity as measured by DPPH and ABTS assays. The authors used a cisplatin-induced nephrotoxicity kidney injury model. Rats with cisplatin-induced nephrotoxicity treated with the purwoceng extract showed an improved kidney function with enhancements in the glomerular score, suggesting nephroprotective and anti-inflammatory effects. Docking analysis from the compounds in the extract showed a strong binding affinity for COX2 and eNOS, suggesting these enzymes are targets for the extract’s anti-inflammatory effect. Although the authors discuss the limited therapeutic range of their extract, since high concentrations induced nephrotoxicity, probably due to the accumulation of compounds, this study suggests that future studies should evaluate the nephroprotective effects of compounds identified as important modulators of important enzymes involved in inflammation such as pinellic acid, and future pharmacokinetic/tissue distribution studies are granted in order to ensure a clinically relevant effect while avoiding toxicity.

### 1.5. Assessing the Anti-Inflammatory and Antioxidant Activity of Mangiferin in Murine Model for Myocarditis: Perspectives and Challenges

In the manuscript by Popa et al. [Contribution 5], the authors evaluate the role of mangiferin, a glucosyl xanthone isolated from *Mangifera indica* with known antioxidant, anti-inflammatory, cardioprotective, antibacterial, antiviral, and immunomodulatory effects, in a myocarditis mouse model. This compound is known to inhibit NF-kB, upregulate TGF-β and to downregulate COX2 transcriptional activity, preventing the expression of inflammatory mediators. Mangiferin increased the total antioxidant capacity in mice with myocarditis. It significantly lowered creatin kinase and aspartate aminotransferase levels and decreased left ventricular thickness compared to the untreated group. These effects were more pronounced than the ones induced by prednisone (the conventional treatment) or Trolox. These effects were attributed to mangiferin’s antioxidant and anti-inflammatory effects since some of the observed effects were mimicked by Trolox, an antioxidant. Mangiferin also significantly reduced serum pro-inflammatory markers. This study supports further functional studies regarding the role of mangiferin for myocarditis treatment to facilitate translational applications.

### 1.6. Exploring the Protective Effects of Taxifolin in Cardiovascular Health: A Comprehensive Review

This review by Sim et al. [Contribution 6] explores the potential use of taxifolin (dihydroquercetin or 3,5,7,3′,4′-pentahydroxyflavanone), a natural flavonoid found in plants such as Siberian larch (*Larix sibirica*, Pinaceae), milk thistle (*Silybum marianum*, Asteraceae), and onions (*Allium cepa*, Amaryllidaceae) in cardiovascular health. Taxifolin is known for its antioxidant, anti-inflammatory, hepatoprotective, anticancer and neuroprotective effects. Its described cellular molecular targets include the activation of antioxidant enzymes, inhibition of inflammation through the NF-kB and PI3K/Akt pathways, cell cycle arrest, apoptosis induction, EGFR modulation, attenuation of amyloid beta accumulation and ROS scavenging. It has also been shown to exhibit antibacterial and antiangiogenic effects. As mentioned in this review, among flavonoids, taxifolin is known as a particularly potent antioxidant, being more effective than quercetin and shown to directly scavenge ROS, activate superoxide dismutase, modulate NRF2/HO-1 and chelate iron ions. The authors describe that most of its effects are attributed to its antioxidant power, related to its anti-inflammatory capacity, but that other molecular targets have also been described such as the PI3K/Akt and TXNIP-NLRP3 pathways, modulating the production of inflammatory mediators and preventing damage in different models. Of relevance for the subject, in cardiovascular health, flavonoid consumption is associated with a reduced risk of CVD and some flavonoid-based drugs have been approved for its treatment. In this regard, taxifolin has been demonstrated to have antihypertensive activity, to lower blood pressure, to have vasorelaxant functions, to decrease cytokine activity in blood vessels, to inhibit vessel wall thickening, to reduce damage to endothelial cells and to induce structural improvements in blood vessels. Described mechanisms include the inhibition of ACE activity, increasing NO bioavailability. Taxifolin has also shown cardiomyocyte protective effects in various cardiac injury models, where mechanisms include the regulation of the NRF2/HO-1, ERK, JNK, JAK2/STAT3 and Smad signaling pathways, and to have antihyperlipidemic effects. Thus, as the authors conclude, more clinical and pharmacological studies are needed to generate data on safety, dosage and possible side-effects to support the use of taxifolin for CVD treatment.

### 1.7. Research Progress on Anti-Inflammatory Mechanism of Inula cappa

In the review by Wu et al. [Contribution 7], the authors explore the potential use of *Inula cappa* (also known as Zhuerfeng, Yangerfeng or Shanbaizhi), a natural Chinese herbal subshrub found in tropical and subtropical regions belonging to the Asteraceae family, in inflammatory diseases. *Inula cappa* is known for its anti-inflammatory, antibacterial, antioxidant, hepatoprotective and analgesic effects. Its described cellular molecular targets include the modulation of inflammatory transduction pathways such as MAPK, NF-κB, and TLR2/MyD88, leading to the regulation of pro-inflammatory cytokines such as TNF-α, IL-6, IL-1β, and NO. It has also been shown to exhibit immunomodulatory, detumescent, and free radical scavenging effects. As mentioned in this review, among ethnomedicinal plants, *Inula cappa* is known as a natural potent anti-inflammatory agent, containing key bioactive components such as luteolin, chrysoerilol, artemetin, chlorogenic acid, neochlorogenic acid, cryptochlorogenic acid, isochlorogenic acids A, B, and C, and 1,3-O-dicaffeoylquinic acid, and shown to directly reduce xylene-induced ear swelling, attenuate acetic acid-induced capillary permeability, suppress cotton ball granuloma formation, and inhibit LPS-stimulated NO release. The authors describe that many of its described effects are attributed to its antioxidant power, related to its anti-inflammatory effects, but other molecular targets have been described, including ERK, JNK, and p38MAPK cascades, modulating the production of inflammatory mediators and preventing tissue damage in different pneumonia and osteoarthritis models. Of relevance for the subject, in inflammatory conditions, herbal medicine consumption is associated with a reduced risk of chronic inflammatory disorders. Thus, *Inula cappa*-based formulations such as Juhuang Shangqing Tablets, Pearl Dropping Pills, and Shuanghou Bitong Granules are regularly used in traditional Chinese medicine. In this regard, *Inula cappa* has been demonstrated to have anti-exudative activity, improve local blood circulation, have tissue repair functions, decrease cytokine activity in inflammatory cells, inhibit arachidonic acid metabolic enzymes, reduce damage to endothelial and pulmonary cells, and induce structural improvements in inflamed tissues. Described mechanisms include the inhibition of TLR2/MyD88 complex formation and IκB degradation, preventing p65/p50 NF-κB translocation into the nucleus. *Inula cappa* active constituents have also shown cellular protective effects in various inflammatory injury models (such as *Klebsiella pneumoniae*-induced pneumonia and LPS-stimulated macrophages), where mechanisms include the regulation of the Nrf2/HO-1, p38MAPK, JNK, ERK, and TLR2/MyD88/NF-κB signaling pathways, and to have metabolic and antipyretic effects. Thus, as the authors conclude, more clinical and pharmacological studies are warranted to generate data on safety, dosage and possible side-effects to support the use of *Inula cappa* for inflammatory disease treatment since not all the mechanisms described for the isolated compounds found in this plant can automatically be attributed to *Inula cappa*.

### 1.8. Potential Use of Selected Natural Compounds with Anti-Biofilm Activity

The rise of antibiotic resistance is critical and globally worrisome; these worries are increased by the capacity of microorganisms to develop biofilms. These structures protect the bacteria within an extracellular matrix and communication system (quorum sensing), increasing its resistance up to 1000 times in comparison with the planktonic forms. Approximately 65% of all the bacterial infections are associated with the formation of biofilms. These structures are commonly found in infections of the urinary track (ITU), otitis, endocarditis, osteomyelitis, and pneumonia, as well as in medical devices (prostheses, catheters, and pacemakers).

The review by Fydrych et al. [Contribution 8] synthetized the research on key phytochemicals—quercetin and quercetin rutinoside, apigenin, arbutin, gallic acid, procyanidins—and vitamin C, as therapeutic alternatives. The main results in the report are indicative of the intrinsic antibacterial capacity of the compounds, but also the capacity of interrupting the integrity of the biofilm structure and the silencing of the bacterial communication. The integration of these phytochemicals in combination with conventional antibiotics and the use of nanotechnologies to overcome the limitations of bioavailability represent the forefront of the strategies using anti-biofilm treatments of chronic infections, including those of the urinary track, respiratory track, and soft tissues.

There are several innovations for the delivery of synergic therapeutics, including the development of silver nanoparticles (AbNPs) to encapsulate flavonoids (e.g., quercetin) in order to improve the anti-biofilm effect in diabetic and burns wound healing. On the other side, the use of hydrogels of xanthan or chitosan with gallic acid or rutin (quercetin rutinoside) allows a controlled release and extended presence in the infection site. Nonetheless, nano emulsions are used to improve the bioavailability of rutin (by 34 times) after oral administration.

The combination strategy using phytochemicals and antibiotics may allow several advantages, including the resensibilization (the phytochemicals may recover the efficacy of antibiotics against multi-resistant strains (MDR)), and/or the reduction in toxicity (lower dose of chemotherapeutics, reducing nephrotoxicity or neurotoxicity (e.g., using colistin).

As a conclusive remark, the integration of phytochemicals in the clinical practice offers a promising way to fight the antimicrobial resistance, because of its capacity of blocking the bacterial quorum sensing (QS) and preventing the cell adhesion, and therefore, the potential healing and prophylactic capacity of these compounds may have great potential for the future.

## 2. Conclusions

Research on bioactive compounds has provided valuable information on new drug design for the treatment of several highly prevalent diseases. Solid and further pharmacological and clinical research is warranted to ensure their timely translation and application.

From the point of view of suggesting a ‘take-home lesson’ for future steps in the research of molecular aspects of health promotion with natural products, using the example of the botanical preparation POW9™ as a study type, as well as the work with phytochemicals for anti-quorum sensing effects or purwoceng for the prevention of kidney damage, there are many open questions to pursue or actions to take in terms of validation. Future research should investigate the stability of the formulations, the optimization of a bioactivity-guided-fractionation strategy, and/or the potential of improving the delivery of the bioactives in target cells (e.g., by (nano)encapsulation). In particular, scholars must make a deeper commitment to the demonstration of bioaccessibility, bioavailability, metabolism, and biological activity (from in vitro to preclinical and clinical research) in research work, operate at physiologically relevant dosages compatible with human intervention studies, and use the best hints or the selected best-performer biocomponent/s.

The case of the 9-part cocktail is an example where an excessive number of identities may not lead to a significant boosted effect (synergy or additive effects), especially when the dosage for a given result is too high to be compatible with the inclusion in a dietary intervention as coadjutant. Other examples in the same line of thought—concerning antimicrobial activity, as well as the design of personalized therapies that take advantage of the synergism between natural products and conventional chemotherapeutical agents or that revert damage induced by chemotherapy as in the case of mangiferin—should be further supported by multidisciplinary research lines (and teams) integrating chemical, biological, and biomedical research and innovation ventures.

Collectively, these studies highlight the therapeutic significance of natural compounds as multi-target candidates for managing complex chronic conditions, including cardiovascular, neurodegenerative, and systemic inflammatory disorders, and as potential chemopreventive compounds that act against carcinogenesis. The central scientific insight emerging from these articles is their shared pleiotropic mechanism of action: rather than targeting isolated molecular endpoints, these phytochemicals concurrently modulate critical intracellular signaling pathways, notably Nrf2/HO-1, NF-κB, MAPK (ERK, JNK, p38), and TLR2/MyD88. Through these cascades’ regulations, they mitigate oxidative damage by scavenging ROS and enhancing endogenous antioxidant enzymes, downregulate pro-inflammatory cytokines (such as TNF-α, IL-6, and IL-1β), and preserve tissue structural integrity.

Ultimately, these findings establish a strong biochemical rationale bridging natural pharmacotherapy with modern molecular targeted medicine, emphasizing the critical necessity for further translational and robust clinical trials and investigations to establish pharmacokinetic profiles and standardize dosage protocols, evaluate bioavailability, and confirm safety characteristics for therapeutic adoption.

## Abbreviations

The following abbreviations are used in this manuscript:

ABTS	2,2-azino-di-(3-ethylbenzotiazoline)-6-sulfonate acid
ALA	Alpha-lipoic acid
ACE	Angiotensin-Converting Enzyme
B(a)P	Benzo(a) pyrene
BSE	Broccoli sprout extract
CAT	catalase
CVD	Cardiovascular disease
COX	Cyclooxigenase
CSE	cystathionine-γ-lyase
DPPH	2,2-diphenyl-1-picrylhydrazyl
EGFR	Epidermal Growth Factor Receptor
ERK	Extracellular-signal Regulated Kinase
eNOS	Endothelial Nitric Oxide Synthase
JAK2/STAT3	Janus Kinase 2/Signal transducer and activator of transcription 3
JNK	Jun N-terminal Kinase
MAPK	Mitogen Activated Protein Kinases
MDA	malondialdehyde
NLRP3	NLR family pyrin domain containing 3
NO	Nitric oxide
NRF2/HO-1	Nuclear factor erythroid 2-related factor 2/Hemoxigenase-1
PI3K/Akt	phosphoinositide-3-kinase/Protein Kinase B
RCA	Red cabbage leaf aqueous extract
ROS	Reactive Oxygen Species
SOD	Superoxide dismutase
TFC	Total flavonoid content
TGF-β	Transforming Growth Factor β
TPC	Total phenolic content
TXNIP	Thioredoxin-Binding Protein
TEAC	Trolox equivalent antioxidant capacity
